# Truth in the Details 

**DOI:** 10.3201/eid2004.AC2004

**Published:** 2014-04

**Authors:** Sharon Bloom, Emily M. Weeks

**Affiliations:** Centers for Disease Control and Prevention, Atlanta, Georgia, USA (S. Bloom);; Independent Art Historian, New Haven, Connecticut, USA (E.M. Weeks)

**Keywords:** emerging infectious diseases, John Frederick Lewis, On the Banks of the Nile, Upper Egypt, Truth in the details, zoonosis, Middle East respiratory syndrome coronavirus, avian influenza virus, camels, birds, about the cover art

**Figure:**
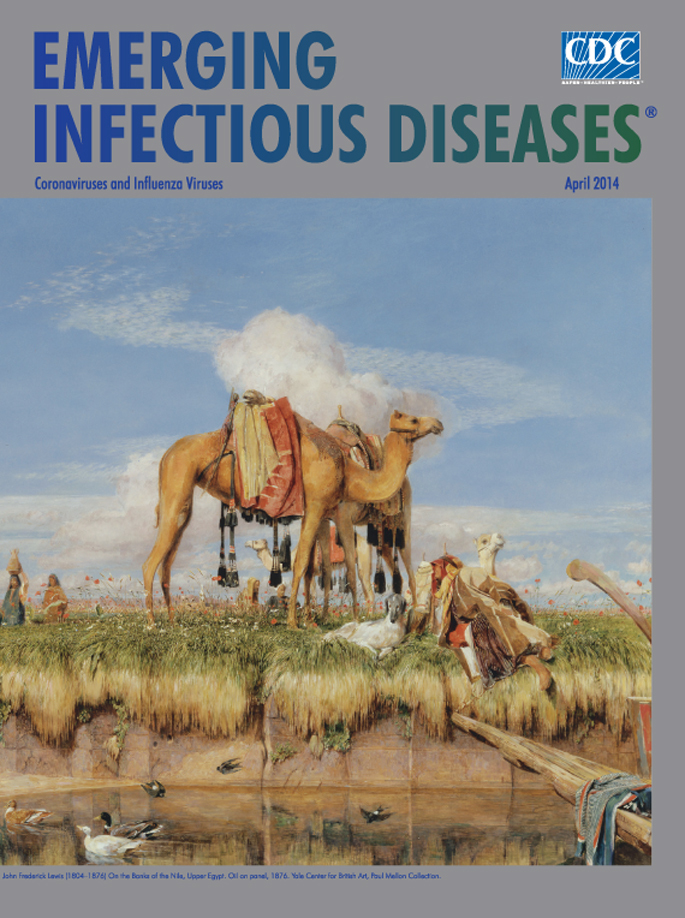
**John Frederick Lewis (1804–1876) On the Banks of the Nile, Upper Egypt. Oil on panel, 1876.** Yale Center for British Art, Paul Mellon Collection. [B1981.25.418]

“Let him examine, for instance, with a good lens, the eyes of the camels, and he will find there is as much painting beneath their drooping fringes as would, with most painters, be thought enough for the whole head . . . ”

—John Ruskin, art critic (1856), regarding the work of John Frederick Lewis.

John Frederick Lewis (1804–1876) was a British painter who specialized in exquisitely detailed paintings of Oriental (Middle Eastern) subject matter, inspired by his ten-year residency in Egypt. Having become an exhibiting member of London’s Old Watercolor Society by 1827, Lewis would later turn almost exclusively to oil painting. His early works favored animals and hunting scenes, but his compositions became increasingly diverse after he had traveled to Spain and Europe. In 1841 his travels took him to Cairo, where he remained until 1851, making numerous drawings and gathering the materials that would confirm his growing reputation in London as an “artist-ethnographer.”

*On the Banks of the Nile, Upper Egypt,* painted shortly before Lewis’s death in 1876, depicts a springtime scene in rural Egypt, in which a group of traveling Bedouin and their camels pause to meet the local *fellaheen* (peasant farmers). One Bedouin man and his saluki hunting dog rest at the edge of the river, beneath the towering form of a camel, silhouetted against the sky. True to Lewis’s realistic style, the details of the man’s clothing are rendered with nearly photographic accuracy; he wears the traditional *kufiyeh* (head scarf) and a heavy outer garment of wool and cotton, its voluminous folds showing off a distinctive pattern of broad brown and cream panels. Also typical of the artist’s work is the easy parlance between man and beast: the camels wait contentedly for their riders to return, while the dog and waterfowl adopt similarly relaxed demeanors. In the distance are female peasants, who traverse a field of orange and white wildflowers on their way to fetch water from the Nile. They are as much a part of the landscape as are the flora, and they provide a gentle reminder of Lewis’s fascination with beauty of all kinds. The viewpoint that Lewis offers—that of an unacknowledged observer upon an idling boat—seems to suggest that we too belong in his composition, as unobtrusive additions to this delicately balanced natural world.

Several Orientalist motifs of Lewis’s art are mirrored in this month’s issue of Emerging Infectious Diseases. Lewis’s juxtaposition of travelers, wild birds, and domesticated animals illustrates ideal opportunities for disease transmission. For example, since 2012, Middle East respiratory syndrome coronavirus (MERS-CoV) has caused an ongoing outbreak of severe acute respiratory tract infection in humans; new findings add to the growing evidence that MERS-CoV, or a closely related virus, infected dromedary camels in the United Arab Emirates long before the first human case of MERS-CoV. While the specific role of camels in MERS-CoV transmission remains unclear, the camel mystery deepens with a report of a novel coronavirus of camels, related to but distinct from MERS-CoV. Wild waterfowl similar to those vividly portrayed by Lewis play a key role in the transmission of avian influenza viruses; active surveillance of avian influenza viruses among domestic poultry in Egypt found that subtype H5N1 still circulates in a widespread manner and that subtype H9N2 is emerging.

The words of art critic John Ruskin, quoted above, allude to Lewis’s artistic mission and provide an unexpected link between the disciplines of art and science: In Lewis’s meticulous attention to detail and in the careful research of this month’s scientists, we witness the tireless pursuit of truth.
